# Constrained Spherical Deconvolution White Matter Tractography in Neuro-Oncology and Deep Brain Stimulation: An Illustrative Case Series

**DOI:** 10.3390/brainsci16050501

**Published:** 2026-05-02

**Authors:** Francesca Romana Barbieri, Massimo Marano, Daniele Marruzzo, Alessandra Ricci, Brunetto De Sanctis, Alessandro Riario Sforza, Riccardo Paracino, Stefano Toro, Serena Pagano, Fabrizio Mancini, Carolina Noya, Davide Luglietto, Riccardo Antonio Ricciuti

**Affiliations:** 1 Department of Neurosurgery, Azienda Ospedaliero-Universitaria Sant’Andrea, 00189 Roma, RM, Italy; francesca.barbieri87@gmail.com; 2 Research Unit of Neurology, Neurophysiology, Neurobiology and Psychiatry, Department of Medicine, Università Campus Bio-Medico di Roma, 00128 Rome, RM, Italy; stefano.toro@unicampus.it; 3 Fondazione Policlinico Universitario Campus Bio-Medico, Viale Alvaro del Portillo 200, 00128 Rome, RM, Italy; 4 Department of Neurosurgery, Ospedale Santa Rosa, 01100 Viterbo, VT, Italy; daniele.marruzzo@yahoo.it (D.M.); s.pagano.med@gmail.com (S.P.); 5 Department of Physics, Ospedale Santa Rosa, 01100 Viterbo, VT, Italy; alessandra.ricci@asl.vt.it; 6 Department of Psychology, ASL Viterbo, 01100 Vitebro, VT, Italy; brunetto.desanctis@asl.vt.it; 7 Department of Radiology, Ospedale Santa Rosa, 01100 Viterbo, VT, Italy; alessandro.riariosforza@asl.vt.it; 8 Department of Neurosurgery, Ospedale Santa Maria della Misericordia, 06129 Perugia, PG, Italy; r.paracino@gmail.com (R.P.); fabrizio.geremia.mancini@gmail.com (F.M.); 9 Department of Neurosurgery, Azienda Ospedaliera San Camillo Forlanini, 00100 Rome, RM, Italy; carolinanoya1@gmail.com (C.N.); davidelugliettomd@gmail.com (D.L.); riccardo.ricciuti@gmail.com (R.A.R.)

**Keywords:** constrained spherical deconvolution, tractography, neuro-oncology, deep brain stimulation, diffusion MRI, white matter pathways

## Abstract

**Highlights:**

**What are the main findings?**

- Constrained spherical deconvolution (CSD) tractography outperformed diffusion tensor imaging in visualizing eloquent white matter pathways in complex neurosurgical settings.
- CSD-based reconstructions reliably matched intraoperative stimulation findings and postoperative functional outcomes in neuro-oncology and DBS cases.

**What are the implications of the main findings?**

- CSD tractography may improve surgical safety by refining functional boundaries during tumor resection in eloquent brain regions.
- Personalized CSD-guided planning can optimize DBS targeting and stimulation programming, supporting its integration into routine neurosurgical workflows.

**Abstract:**

**Background/Objectives**: Preservation of critical white matter (WM) pathways is essential for maximizing surgical safety in neuro-oncology and functional neurosurgery. Constrained spherical deconvolution (CSD) offers superior modeling of complex fiber architecture compared to diffusion tensor imaging (DTI). This case series evaluates the clinical utility of CSD in surgical planning and intraoperative navigation. **Methods**: A retrospective review of 20 patients (15 brain tumors, 5 functional disorders) treated between September 2022, and September 2024 was performed. All patients underwent preoperative MRI with CSD-based reconstruction of eloquent WM tracts. Clinical presentation, tract involvement, surgical strategy, and postoperative outcomes were analyzed. **Results**: CSD reliably reconstructed CST, AF, IFOF, OT, and DRTT depending on tumor location or DBS target. Compared with standard DTI, CSD provided improved delineation of tract extent and tumor–tract interfaces. Gross total resection (GTR) was achieved in all tumor patients without new neurological deficits. DBS cases showed precise correlation between stimulation thresholds, side effects, and CSD-predicted distances to critical WM tracts. DRTT targeting resulted in marked clinical improvement in Holmes tremor. **Conclusions**: CSD enhances anatomical accuracy in WM tract visualization, supporting safer resections in eloquent areas and improving DBS targeting. Its integration into routine workflow may optimize neurosurgical outcomes.

## 1. Introduction

Preserving neurological function while achieving the maximal therapeutic benefit is a main goal of resective and functional modern neurosurgery. This balance becomes extremely relevant when lesions or surgical targets lie in or near eloquent cortical and subcortical regions and relative white matter tracts. For neuro-oncological patients, the resection of diffuse gliomas and other infiltrative tumors must be planned with an accurate understanding of the tumor—white matter (WM) interface to reduce the risk of collateral lesions. Achieving the gross total resection (GTR) remains a cornerstone of improved survival in glioma surgery; however, resection must be tailored to functional boundaries rather than purely anatomical ones. This paradigm shift has increased the importance of preoperative functional imaging techniques that can complement intraoperative mapping [1,2,3,4]. Likewise, in functional neurosurgery and deep brain stimulation (DBS), millimetric precision is required to avoid adverse effects of stimulation while maximizing therapeutic benefit. Classical stereotactic approaches rely on atlas-based coordinates and structural magnetic resonance imaging (MRI), but these methods cannot fully reveal interindividual variability in WM architecture. Understanding the proximity of electrodes to pathways such as the corticospinal tract (CST), optic tract (OT), or tracts connecting basal ganglia and cerebellum is increasingly recognized as crucial for DBS surgical planning, stimulation programming and long-term outcomes [5].

Diffusion tensor imaging (DTI) has historically been used to map WM organization, but its assumption of a single dominant fiber orientation per voxel inherently limits its applicability. More than 90% of WM voxels contain complex fiber geometries, which are not adequately represented by DTI. These limitations become even more pronounced near tumors, where edema, mass effect, and tract displacement further complicate diffusion modeling. Consequently, DTI-based reconstructions frequently underestimate tract extension, particularly in regions where fibers cross or diverge [6].

Constrained spherical deconvolution (CSD) overcomes these limitations by estimating a fiber orientation distribution (FOD) that models multiple intravoxel orientations. This allows for a more accurate depiction of WM architecture, especially in areas previously inaccessible to reliable analysis using DTI. CSD has shown advantages in representing associative tracts such as the arcuate fasciculus (AF) and inferior fronto-occipital fasciculus (IFOF), as well as projection pathways like the corticospinal tract (CST), and cerebellothalamic pathways such as the dentato-rubro-thalamic-tract (DRTT) [7].

In this case series, we illustrate the clinical application of CSD in a heterogeneous cohort including patients with brain tumors located in eloquent areas and individuals undergoing DBS for movement disorders. We aim to highlight the practical impact of CSD on preoperative planning, intraoperative decision-making, surgical safety, and early postoperative outcomes.

## 2. Materials and Methods

### 2.1. Study Design

This is a retrospective report of a case series of 20 consecutive patients undergoing awake neurosurgery between September 2022 and September 2024 at the Neurosurgery Unit of the Santa Rosa Hospital of Viterbo. Fifteen patients with intracranial tumors were collected in the “neuro-oncology” group, while five more patients undergoing DBS for Parkinson’s disease (PD), dystonia or tremor were categorized as belonging to the “movement disorders” group. All subjects underwent routine preoperative neurological and surgical evaluation for symptom assessment, and MRI acquisition suitable for CSD reconstruction and neuronavigation. Finally, all of them received awake surgery with intraoperative neurophysiology monitoring (IONM) with or without direct electrical stimulation (DES). Due to the small sample size and marked heterogeneity between patients and surgical indications, no formal statistical comparison between CSD and DTI was performed. All quantitative observations should therefore be interpreted as exploratory.

### 2.2. Imaging and Tractography

All the MRIs were performed with a 1.5 T Achieva (Philips Healthcare, Amsterdam, Noord-Holland, The Netherlands, R5.3.0.3) MR machine using a 16-channel neurovascular coil. MRIs were acquired using standardized DTI protocols optimized for CSD. DTI series were used for fiber tracking, with gradients applied in 15 different directions and two b factor (b = 0 s/mm^2^ and b = 1000 s/mm^2^). Acquisition was made in the axial plane with no angulation (the diffusion directions in the DICOM headers are not rotated when the scan is acquired obliquely, so no angulation is allowed) and contiguous (with no-gaps) slices with a spacing and a thickness of 2 mm were performed. Other MRI sequences were collected including morphological scans (axial 3D fluid-attenuated inversion recovery (FLAIR) [8], axial gradient echo (GRE), 3D T1 weighted and an ultrafast GRE scan including a magnetization-preparation with turbo field echo (TFE) after intravenous contrast administration).

The adoption of 3D FLAIR scans allowed thinner slices with multi-planar reformation capability, a higher flow sensitivity, a higher sensitivity to subtle T1 changes in fluid, no cerebrospinal fluid (CSF) inflow artifacts, and a better visualization of WM abnormalities to be achieved, providing a 3D dataset compatible with computer-aided analysis.

Tractography was performed on StealthStation™ S8 (Medtronic, Chicago, IL, USA) after rigid co-registration of diffusion and anatomical images. Reconstruction parameters were manually optimized for each patient. The quantitative anisotropy (QA) threshold ranged from 0.04 to 0.08 depending on signal quality and tract complexity. Angular threshold ranged between 45° and 60°. Streamline number was initially set between 100 and 300 fibers and then adjusted to improve tract continuity while avoiding anatomically implausible projections. Tractography was performed using deterministic propagation constrained by CSD-derived fiber orientation distributions. ROI placement followed standardized anatomical landmarks for each tract. For CST reconstruction, ROIs were positioned in the cerebral peduncle, posterior limb of the internal capsule, and precentral gyrus. For AF and IFOF, ROIs were placed according to known frontal and temporo-parietal landmarks. DRTT reconstruction was obtained through ROIs in the dentate nucleus, superior cerebellar peduncle, and thalamic target region. Quantitative tractography metrics were retrospectively extracted when available, including streamline count, estimated tract volume, and minimal distance between reconstructed tracts and surgical targets. In cases undergoing direct subcortical stimulation, the estimated distance between the reconstructed corticospinal tract and the stimulation site was compared with stimulation thresholds recorded intraoperatively.

Moreover, we acknowledge that the diffusion acquisition protocol used in this retrospective series (15 diffusion directions, b = 1000 s/mm^2^) is suboptimal for modern CSD reconstruction. However, this satisfies the mathematical requirement for a spherical harmonic expansion. Furthermore, the non-negativity constraint inherent in the CSD algorithm enables ‘super-resolution,’ allowing the model to resolve complex fiber orientations that a single-tensor DTI model inherently underestimates. The clinical validity of this approach is supported by our intraoperative findings, which demonstrated a high correlation between CSD-derived tract projections and direct subcortical stimulation.

### 2.3. CSD Reconstructions

**Figure 1:**
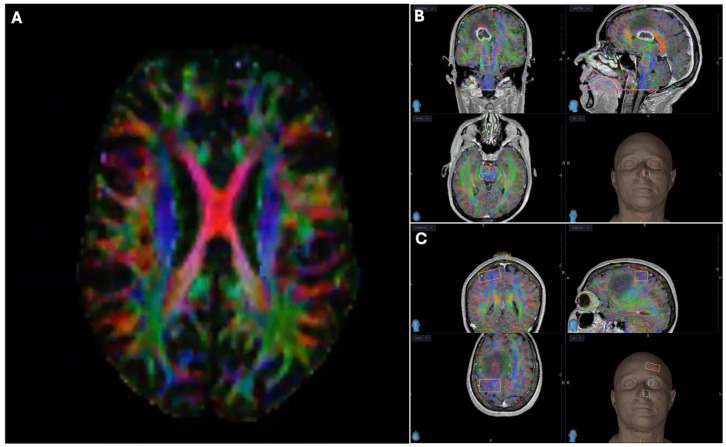
(**A**) DEC map showing fiber directions: red for right-left, green for antero-posterior, and blue for superior-inferior direction; (**B**) Volumetric T1 scan fused with DEC map showing bundle reconstruction through the detection of an initial (e.g., for CST in brainstem), if necessary an intermediate (e.g., for CST in the internal capsule) and (**C**) a final ROI (e.g., for CST in the motor cortex). Rectangles, ROIs.

Reconstruction of CSD for preoperative surgical planning and for intraoperative use was performed on StealthStation™ S8 (Medtronic, Chicago, IL, USA). Once the images were transferred to the StealthStation™ system (Medtronic, Chicago, IL, USA) we evaluated the accuracy of the MRI acquisition parameters and we checked the merge fusion. The fusion could be made by linking DTI to either T1 or T2 scans. The directionally encoded color (DEC) map showed fiber directions: red for right-left, green for antero-posterior, and blue for superior-inferior direction (Figure 1A).

Bundle reconstruction was performed through the detection, on a volumetric T1 scan, of an initial region of interest (ROI) (e.g., for CST in brainstem), an intermediate ROI (e.g., for CST in the internal capsule) and a final ROI (e.g., for CST in the motor cortex) (Figure 1B,C). Once the bundle of interest was reconstructed, it was possible to modify it, removing unnecessary ROIs to make the reconstruction even more accurate and specific. To increase accuracy, it would be recommended to use the DEC map, in addition to T1 and T2 scans.

**Figure 2:**
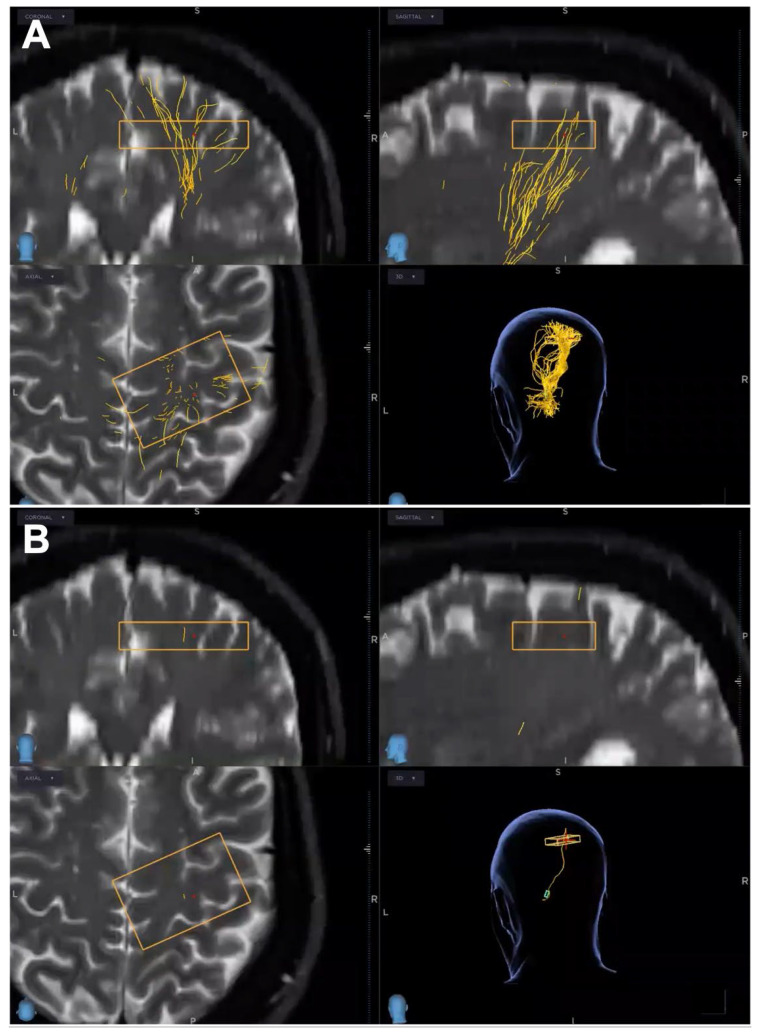
Parameters editing during the preoperative reconstruction and planning phase. (**A**) QA threshold reduction leads to a sensitivity increase, consequently getting more fibers and (**B**) QA threshold increase leads to a specificity increase, consequently getting less fibers. Rectangles, ROIs.

During the preoperative reconstruction and planning phase, it was possible to edit some parameters, namely the number of fibers and quantitative anisotropy (QA) threshold, to increase sensitivity or specificity. More specifically, the QA threshold represents the probability level that must be exceeded for fibers to continue propagating. Lowering this threshold increases sensitivity by allowing a greater number of fibers to be included, whereas raising the threshold increases specificity by restricting propagation to fewer fibers (Figure 2A,B).

By increasing or decreasing the number of fibers, we can increase sensitivity or specificity, respectively. Additionally, increasing the CSD angle—the angle along which the fibers propagate—allows for improved angular resolution.

Additional procedures for DBS neuronavigation and planning, and intraoperative monitoring are reported in Appendix A.

### 2.4. Post-Operative Imaging

All subjects received post-operative neuroimages. Neuro-oncology cases underwent MRI with contrast agents (gadolinium) for evaluating the GTR and the integrity of the adjacent white matter tracts.

DBS cases received a postoperative volumetric 0.6 mm slices CT scan for achieving tridimensional reconstruction of electrodes through the SureTune™ 4 software (Medtronic, Chicago, IL, USA), enabling the creation of patient-specific anatomy, lead location and orientation.

Institutional Review Board approval and patients’ consent form were not required, as the study is a retrospective case series. All procedures performed were in accordance with the local Ethical Committees and with the 1964 Helsinki declaration and its later amendments or comparable ethical standards.

## 3. Results

### 3.1. The Neuro-Oncology Series

The neuro-oncology group included patients with intracranial cancers of eloquent cortical and subcortical regions. The group was made up by nine women and six men, the average age was 60 (range 18–80, SD = ±18.95). Seven of them will later receive a histological diagnosis of GBM (WHO grade IV), three of Oligodendroglioma with 1p-19q deletion, IDH mutation (WHO grade III), three of Oligodendroglioma with 1p-19q deletion, IDH mutation (WHO grade II), and two of lung adenocarcinoma metastasis. Clinical presentations varied widely, reflecting the functional specialization of the affected pathways. Seizures were common in patients with temporo-insular or frontal lesions, whereas motor deficits predominated in those with fronto-parietal tumors. Tumors in the dominant hemisphere frequently caused speech impairments, while parieto-occipital lesions were associated with visual field disturbances, particularly homonymous hemianopsia (Table 1).

**Figure 3:**
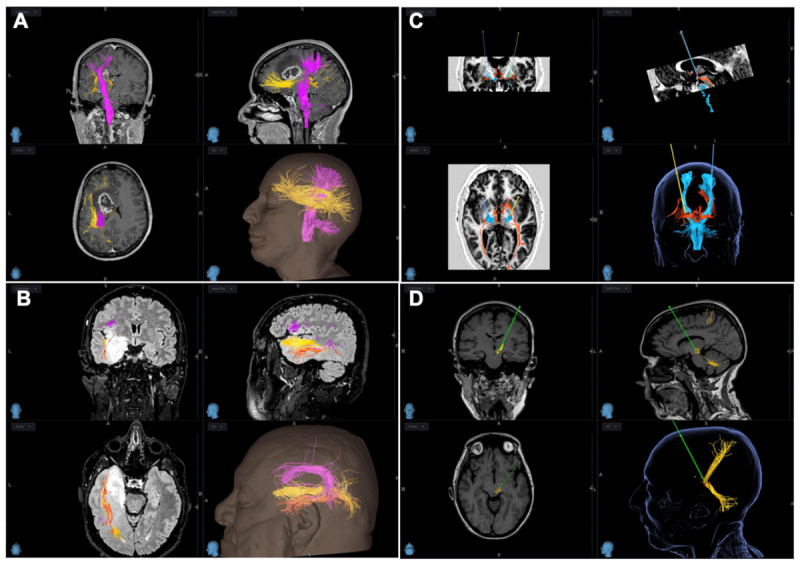
Main white matter tracts reconstructed through CSD. (**A**) CST in pink and AF in yellow, (**B**) AF and IFOF In red, (**C**) OT in red, CST in blue and (**D**) DRTT in yellow. Planned lead trajectories are also illustrated in (**C**) (yellow and blu lines) and (**D**) (green line).

The main WM tracts reconstructed through CSD were CST in 15/15 neuro-oncology cases (Figure 3A), AF and IFOF in left temporo-insular (3/15) and left frontal (4/15) lesions (Figure 3A,B), and OT in parieto-occipital (1/15) cancer.

**Figure 4:**
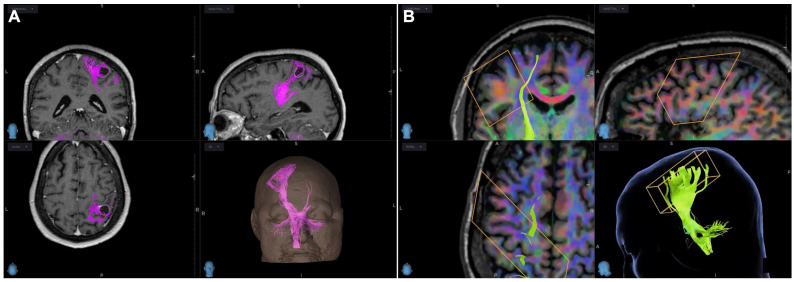
CST reconstruction on neuronavigation system showing accuracy of (**A**) CSD (pink) and (**B**) DTI (green). Rectangles, ROIs.

CSD reconstructions provided a more detailed visualization of white matter tracts than traditional DTI, owing to their ability to depict a broader extent of tract projections toward the corresponding cortical regions (Figure 4A,B).

**Figure 5:**
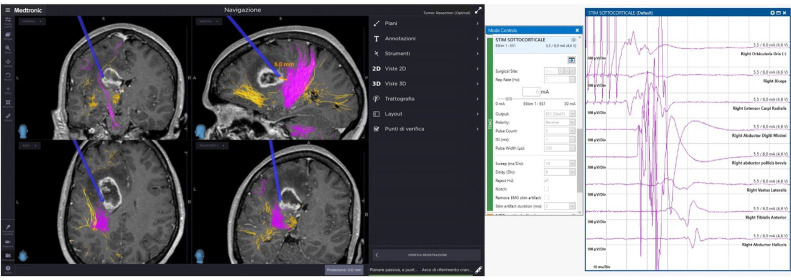
Intraoperative matching between intensity of direct subcortical stimulation (DSCS) and tract reconstruction (CST, pink) distance.

In line with this, intraoperative correspondence between direct subcortical stimulation (DSCS) and tract visualization through neuronavigated CSD reconstructions was accurate and consistent across all cases. Specifically, during tumor resection, the DSCS intensity correlated with the estimated distance from the corticospinal tract (CST) as displayed on the neuronavigation system (Figure 5).

**Figure 6:**
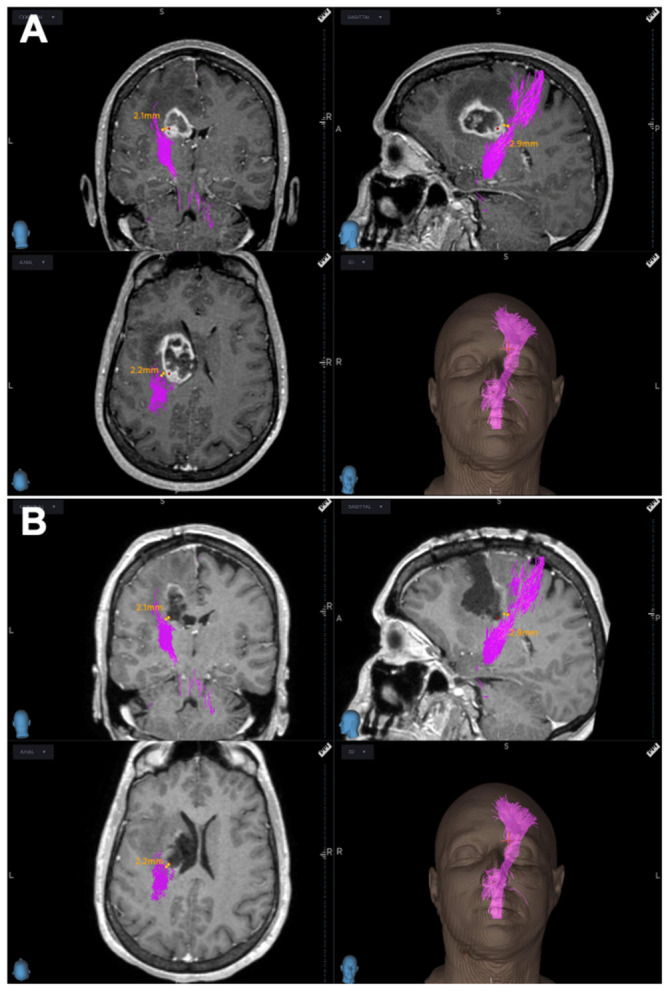
The same distance (in millimeters, mm) between the posterior margin of the tumor and CST (pink) was detected both (**A**) intraoperative and (**B**) on post-operative MRI, showing the lack of intraoperative brain-shift effect on CTS reconstructions.

Moreover, the brain shift observed after tissue removal did not alter the distance between the posterior tumor margin and the CST. The same distance was confirmed both intraoperatively and on the postoperative MRI (Figure 6A,B). Although no evident discrepancy between the tumor margin and reconstructed CST was visually observed after resection, intraoperative brain shift was not formally quantified nor corrected using dedicated intraoperative imaging techniques. The comparison was based only on qualitative assessment between neuronavigation findings and postoperative MRI. Therefore, the potential effect of brain shift on tract location remains a limitation of the present study.

In the neuro-oncology cohort, CSD-based tractography played a central role in defining the optimal surgical corridors. 

In all 15 cases, CST reconstruction through CSD was qualitatively superior in details and anatomical coherence compared with traditional DTI. This was particularly evident in regions where tract geometry becomes more complex (i.e., corona radiata, centrum semiovale, and near the superior longitudinal fasciculus). The enhanced visualization allowed the surgical team to better anticipate areas where functional boundaries might be narrow or where tumor infiltration could distort normal anatomy. 

In dominant-hemisphere tumors, CSD reconstruction of language-associated pathways such as AF and IFOF allowed for a more nuanced understanding of how the lesion interacted with these networks. This supported decisions regarding awake mapping and informed preoperative predictions of potential postoperative language deficits. In multiple cases, AF projections were shown to be displaced rather than infiltrated, encouraging more aggressive but safe resections.

During awake craniotomy, direct cortical and subcortical stimulation (DCS/DSCS) provided real-time validation of tractography findings. The correlation between CSD-predicted tract distances and stimulation thresholds was consistent across all cases. Notably, even in tumors producing substantial edema or mass effect, intraoperative mapping confirmed that CSD accurately depicted tract displacement without introducing false positives. In cases involving the parieto-occipital region, CSD enabled precise identification of the optic radiation, which guided resection planes and likely contributed to postoperative improvement in visual symptoms in the affected patient.

Gross total resection was achieved in every case. No patient developed new postoperative neurological deficits. Many patients experienced meaningful improvements, such as regression of hemiparesis or restoration of speech fluency, suggesting that CSD-guided planning may have contributed to functional preservation.

### 3.2. The Movement Disorders Series

The movement disorders group included three patients with PD, one patient with cervical dystonia and one with Holmes tremor, two women and three. Average age was 48.4 (range 21–63, SD = ±16.46).

Selected DBS targets were bilateral subthalamic nucleus (STN; n = 2 PD), bilateral globus pallidus pars interna (GPi; n = 1 PD, n = 1 CD), and unilateral DRTT (n = 1, tremor).

The main WM tracts reconstructed through CSD were CST in all 5 cases (Figure 3A), OT in 2/5 cases treated with bilateral GPi DBS (Figure 3C), DRTT in 1/5 cases (the Holmes tremor case) (Figure 3D).

**Figure 7:**
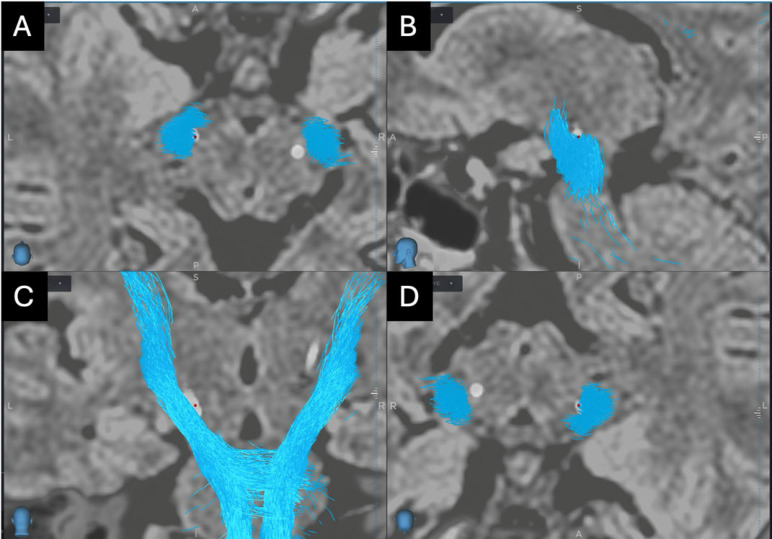
CST (blue) and reconstructed through CSD and electrode positioning. (**A**) axial view, (**B**) sagittal view, (**C**) coronal view, (**D**) probe’s eye view showing lead placement on the STN border, with part of the lead within the right CST.

CSD reconstruction delineated CST and OT in proximity to planning trajectories, improving estimated lead location. Postoperative CT-tridimensional reconstruction showed correct placement of electrodes with no complications in all patients treated with DBS. However, Case 19 reported stimulation side effects due to involvement of right CST, improved following the adoption of directional stimulation (Figure 7). In a patient with Holmes tremor (case 20), CSD enabled visualization of the DRTT, informing a tailored trajectory [8].

In the patient with Holmes tremor, CSD enabled reconstruction of the dentato-rubro-thalamic tract and contributed to trajectory planning. However, because this observation derives from a single illustrative case, no general conclusions can be drawn regarding the clinical value of CSD-guided DRTT targeting.

## 4. Discussion

This expanded case series illustrated the application of CSD-based tractography across a broad range of neurosurgical scenarios. CSD application improved preoperative understanding of white matter anatomy, refined intraoperative decision-making, and contributed to safe postoperative outcomes. This case series supports the growing evidence on the use of advanced tractography into standard neurosurgical workflows, especially when operating within or near eloquent networks.

DTI has long been employed for presurgical mapping, yet its limitations in regions of complex fiber architecture are well recognized. More than 90% of WM voxels contain crossing fibers, rendering the single-tensor model insufficient for resolving true fiber orientation. As also outlined in our series, DTI may underestimate the extent of tracts such as the CST and language-associated pathways like AF and IFOF. By contrast, CSD consistently revealed the complete cortical fan-out and more detailed course of these pathways.

The majority of studies uses the DTI to estimate WM fiber orientations from DWI data [9] and several authors compared the use of DTI with IONMs for the surgical removal of tumors [10,11,12,13] with good concordance [14,15,16,17,18]. But most of them have often pointed out the main limitation of this technique, regarding its reliability to correctly model diffusion signal in different conditions [19,20] and the difficulty of a correct tract reconstruction in some cases, i.e., crossing fibers or fibers close to tumor margins or tumor edema [21]. In fact, it is clear that more than 90% of imaging voxels in the WM contain multiple fiber populations [22]. Accordingly, even large fiber bundles, such as the CST, cannot be adequately represented by DTI-based tractography methods in regions where the tract crosses other major fiber bundles, such as the corpus callosum or the longitudinal fasciculus. In fact, since DTI-based tractography does not consider voxels with low Fractional Anisotropy (FA) values, the quantitative analysis might provide the erroneous information of not significant WM alterations [23]. Moreover, partial qualitative and quantitative information regarding well-known undetected WM bundles such as the lateral portion of CST as well as the anterior portion of AF can lead to a worse, inaccurate or partial reconstruction of these bundles [24,25].

These limitations have been successfully overcome by techniques like High Angular Resolution Diffusion-weighted Imaging (HARDI) [26], Q-Ball Imaging (QBI) [27], and Diffusion Spectrum Imaging (DSI) [28]. CSD is an HARDI modified approach [26] that provides an accurate representation of the fiber orientation and distribution within each imaging voxel. CSD does not require very long acquisition time with respect to DSI [29] and it is able to improve angular resolution if compared to QBI [29].

In one study it is clearly shown how DTI-based tractography methods failed to identify the real and correct cortical extension of CST to the sensorimotor cortex, identifying a narrow subset of tracts extending, in most cases, medially to the vertex. In contrast, the CSD-based tractography method consistently reproduced the expected configuration of CST extending to the entire sensorimotor cortex [21]. As previously shown, these clear and important difference have also been shown in our series.

Furthermore, Farquharson et al. also underline how CSD has been shown to be superior, specifically in providing improved estimates of safety margins for neurosurgical procedures, leading to a more tailored approach and effective removal, at the same time reducing the risk of postoperative neurological deficits [20].

In the previous single report of CSD application in a DBS procedure for Holmes tremor, targeting DRTT, we showed an excellent outcome at 6 months [7]. This case, that to our knowledge was the first described in the literature, and is included in the present series underlined once again the importance and effectiveness of CSD in reconstruction of fiber bundles and in preoperative planning leading to a more tailored approach and to an optimal clinical outcome in a very complex case of disorder of brain connections.

One of the most relevant observations in this series was the concordance between CSD-derived tract location and intraoperative stimulation findings. In tumor surgery, lower subcortical stimulation thresholds corresponded to shorter estimated distances from the corticospinal tract, supporting the spatial plausibility of the reconstructions. However, this relationship was evaluated retrospectively and descriptively and should not be interpreted as a formal validation study.

Despite these encouraging findings, several limitations must be acknowledged. First, the study included a relatively small and heterogeneous cohort composed of two distinct patient populations, namely neuro-oncology and DBS cases. These groups differ substantially in terms of pathology, surgical targets, and outcome measures. Consequently, the present study should be regarded primarily as an illustrative case series.

Second, the diffusion MRI acquisition protocol was suboptimal for modern CSD reconstruction. The use of only 15 diffusion directions and b = 1000 s/mm^2^ likely reduced angular resolution and may have affected the reliability of fiber orientation estimation. Contemporary CSD protocols generally require at least 30–60 directions and, ideally, multi-shell acquisitions. Therefore, our findings should be interpreted as evidence of the feasibility of integrating CSD into a routine clinical workflow rather than as proof of superiority over DTI.

Third, CSD itself has intrinsic limitations. Reconstruction quality depends heavily on the quality of diffusion data and may be adversely affected by motion artifacts, edema, susceptibility distortion, and low signal-to-noise ratio. These issues are especially relevant in patients with movement disorders or large tumors associated with substantial mass effect.

Fourth, surgeons and neurophysiologists were not blinded to the tractography method used during planning and surgery. This lack of blinding may have introduced confirmation bias and potentially amplified the apparent concordance between CSD and intraoperative findings.

Finally, intraoperative brain shift was not quantitatively measured or corrected. The apparent correspondence between preoperative tractography and postoperative imaging was based on qualitative comparison only.

Future prospective studies should employ optimized diffusion protocols, quantitative tractography metrics, inter-rater reproducibility analyses, and formal statistical comparison between CSD and DTI. Such studies will be necessary to determine whether CSD provides clinically meaningful advantages over standard diffusion tensor tractography.

## 5. Conclusions

CSD-based tractography appears to be a feasible and potentially useful adjunct for surgical planning in neuro-oncology and DBS. In this retrospective case series, CSD provided anatomically plausible reconstructions that appeared to correspond with intraoperative findings and postoperative outcomes. However, the limited diffusion acquisition protocol, absence of formal quantitative validation, heterogeneous cohort, and potential observer bias substantially limit the strength of these conclusions. Rather than demonstrating definitive superiority over DTI, the present study highlights the potential clinical utility and feasibility of CSD within real-world neurosurgical workflows. Larger prospective studies with optimized acquisition parameters and quantitative validation are required.

## Figures and Tables

**Table 1:** Summary of surgically treated cases and types of white matter tracts reconstructed through CSD.

Case	Group	Sex	Age	Signs/Symptoms	Lesion Location/ DBS Target	Histopathological Diagnosis	White Matter Tract Reconstructed	Outcome
1	N.O.	F	48	Mild speech impairment	Left frontal	WHO grade II Oligodendroglioma (1p-19q deletion, IDH mutation)	CST, AF, IFOF	GTR, speech improvement
2	N.O.	M	18	Seizures	Left temporo-insular	WHO grade II Oligodendroglioma (1p-19q deletion, IDH mutation)	CST, AF, IFOF	GTR, no deficit
3	N.O.	F	75	Left homonymous hemianopsia	Right pariet-occipital	WHO grade II Oligodendroglioma (1p-19q deletion, IDH mutation)	OT	GTR, hemianopsia improvement
4	N.O.	M	57	Moderate left hemiparesis	Right fronto-parietal	WHO grade III Oligodendroglioma (1p-19q deletion, IDH mutation)	CST	GTR, mild left hemiparesis
5	N.O.	F	66	Seizures	Left frontal	WHO grade III Oligodendroglioma (1p-19q deletion, IDH mutation)	CST, AF, IFOF	GTR, no deficit
6	N.O.	F	76	Gait ataxia	Right frontal	WHO grade III Oligodendroglioma (1p-19q deletion, IDH mutation)	CST	GTR, no deficit
7	N.O.	F	80	Mild right hemiparesis	Left frontal	GBM	CST, AF, IFOF	GTR, hemiparesis regression
8	N.O.	F	80	Mild speech impairment, gait ataxia	Left frontal	GBM	CST, AF, IFOF	GTR, speech improvement
9	N.O.	F	52	Right arm and hand motor deficit	Recurrent left fronto-parietal	GBM	CST	GTR, motor deficit regression
10	N.O.	M	68	Left hemiparesis	Right fronto-parietal	GBM	CST	GTR, hemiparesis regression
11	N.O.	M	64	Mild speech impairment	Left temporo-insular	GBM	CST, AF, IFOF	GTR, speech improvement
12	N.O.	M	21	Left hemiparesis	Right thalamic	GBM	CST	GTR, left hemiparesis
13	N.O.	M	69	Seizures	Left temporo-insular	GBM	CST, AF, IFOF	GTR, no deficit
14	N.O.	F	59	Left arm motor deficit	Right fronto-parietal	Lung adenocarcinoma metastasis	CST	GTR, motor deficit regression
15	N.O.	F	67	Right arm motor deficit	Left fronto-parietal	Lung adenocarcinoma metastasis	CST	GTR, motor deficit regression
16	M.D.	M	31	CD	Bilateral GPi	NA	CST, OT	Dystonia improvement, no stimulation side effects
17	M.D.	M	48	PD	Bilateral GPi	NA	CST, OT	Dyskinesia improvement, no stimulation side effects
18	M.D.	M	63	PD	Bilateral STN	NA	CST	Bradykinesia, tremor and rigidity improvement, no stimulation side effects
19	M.D.	M	59	PD	Bilateral STN	NA	CST	Bradykinesia and rigidity improvement, stimulation side effect with left facial pulling
20	M.D.	F	51	HT	Left DRTT	NA	CST, DRTT	Tremor reduction, no stimulation side effects

AF = arcuate fasciculus; CST = cortico-spinal tract; DRTT = dentato-rubro-thalamic tract; F = female; GBM = glioblastoma; GPi = globus pallidus internus; GTR = gross total removal; IFOF = inferior fronto-occipital fasciculus; M = male; NA = not applicable; OT = optic tract; STN = subthalamic nucleus.

## Data Availability

No new data were created or analyzed in this study. Data sharing is not applicable to this article.

## Abbreviations

The following abbreviations are used in this manuscript:

CSD	Constrained spherical deconvolution
MRI	Magnetic Resonance Imaging
DTI	Diffusion tensor imaging
DSI	Diffusion Spectrum Imaging
WM	White matter
DBS	Deep Brain Stimulation
CST	Cortico Spinal Tract
ROI	Region Of Interest

## Appendix A

***Additional procedures for DBS neuronavigation and planning.***

In DBS cases, preoperative MRI was merged with the preoperative volumetric brain computed tomography (CT) scan with the StealthMerge software (StealthStation™ S8, Medtronic, Chicago, IL, USA), to properly identify the skull structures and fiducial markers and for intraoperative frameless neuronavigation (Nexframe, Medtronic, Chicago, IL, USA). Baladin Software was used as a normalized atlas. CT scan acquisition was made in the axial plane, with a field of view (FOV) between 25 and 28 cm, including the whole skull with all fiducials (not covered) plus 3 cm above. Slices were contiguous, not overlying, with thickness of 0.6 mm during the entire acquisition; images were stored in standard DICOM-3 format.

***Intraoperative neurophysiology monitoring (IONM).***

In all cases affected by intracranial tumors IONM including Electrocorticography (ECoG), transcortical motor evoked potentials (TcMEPs) and transcortical sensory evoked potentials (TcSEPs) were performed. Furthermore, motor mapping with phase reversal visualization, direct cortical (DCS) and subcortical stimulation (DSCS) for CST were performed in cases of fronto-parietal localization; language mapping with Penfield method using hand-held bipolar ball tips probe was performed in all temporal, fronto-temporal and temporo-insular tumors.

TcMEPs were performed through “corkscrew” electrodes based on the international 10–20 system. A very high intensity bipolar or multipolar (mainly anodic) stimulation (with stimulus mode in constant voltage and current) in “fast charge” and “slow charge” mode was performed, through trains with interstimulus interval (ISI) from 1 to 9 ms and single pulse duration between 50 and 500 uS respectively. Analysis time was 100 ms.

After craniotomy, following adequate exposure of the cortex, a four (1 × 4) or six-grid (1 × 6) subdural electrode was placed on the cortical surface over the area of the presumed central sulcus (CS). The CS, somatosensory cortex and phase reversal were identified by generating multiple sensory maps by stimulating the contralateral median nerve at a current of 20–35 mA between frequencies of 2.66–4.79 Hz. Mapping continued until the triphasic waveform was generated with a P25 peak directly over the CS, a part of the P20/N30-N20/P30 complex which is either over the pre- or post-CS area.

After localization of the CS, motor mapping of the cortex was performed by using DCS and ECoG. DCS was performed through two methods: a high frequency pulse train (between 300 and 500 Hz) method by using a monopolar anodal hand-held ball tip probe (XOMED, Inc., Jacksonville, FL, USA) with a pulse duration of 500 μs (in most cases a 5 pulse train with ISI of 4 ms at 250 Hz), with stimulation intensity applied between 2 and 30 mA; a slow frequency long pulse Penfield method by using handheld bipolar ball tips probe with a pulse duration of 300–500 μs at a frequency of 50/60 Hz, with initial stimulation intensity of 2.0 mA and increased as needed until a positive response (compound muscle action potential—CAMP) (with a maximum limit of 20 mA). Stimulation was applied for 3 to 5 s at each site. During stimulation, the subdural grid electrodes were positioned close to the stimulation sites to monitor ECoG for the presence of after discharges (ADs). DCS could be performed on request from the subdural grid electrodes too.

Language mapping was performed by the Penfield method, using a hand-held bipolar ball tip probe with a monophasic or biphasic pulse duration of 0.5–1.0 ms at a frequency of 50/60 Hz. The stimulation was applied for 3 to 5 s at each site. The stimulation was initiated at an intensity of 2.0 mA and increased until a positive response (speech arrest/slurring) or ADs were elicited (with a maximum limit of 15 mA for biphasic stimulation). In case of ADs, ice saline (4 °C) was applied immediately to the exposed cortical surface.

During the awake DBS procedures, we performed intraoperative microelectrode recordings (MERs) and subsequent stimulation to guide the placement of directional leads (SenSight™, Medtronic, Chicago, IL, USA) in all cases.
